# IgA Deficiency and Nephrotic Syndrome in Children

**DOI:** 10.3390/ijerph15081702

**Published:** 2018-08-09

**Authors:** Lorenza Di Genova, Stefania Ceppi, Maurizio Stefanelli, Susanna Esposito

**Affiliations:** Pediatric Clinic, Department of Medical and Surgical Sciences, Università degli Studi di Perugia, 06132 Perugia, Italy; lory.digenova@gmail.com (L.D.G.); stefipg22@yahoo.it (S.C.); maurizio.stefanelli@ospedale.perugia.it (M.S.)

**Keywords:** IgA deficiency, monoclonal antibody, nephropathy, nephrotic syndrome, pediatric nephrology

## Abstract

*Background*: Imunoglobulin A (IgA) deficiency (IgAD) is the most common form of primary immunodeficiency in Western countries. There have been several reports on IgAD complicated by glomerulonephritis in adults, but only very few cases of IgAD with nephropathy have been reported in children. We present two cases of IgAD with relapsing nephrotic syndrome in pediatric age. *Case presentation*: A 4-year-old boy and a 2-year-old boy presented with bilateral periorbital oedema and weight gain. The results of laboratory tests revealed IgAD (IgA < 7 mg/dL), normal creatinine, hypoprotidaemia, hypoalbuminaemia, and nephrotic proteinuria. A diagnosis of IgAD and idiopathic nephrotic syndrome was made, and steroid treatment (prednisone 60 mg/mq/day) was started. During steroid tapering, the children experienced several relapses and to obtain a positive outcome they required therapy with human monoclonal anti-CD20 antibodies (rituximab in the first child, ofatumumab in the second one). *Conclusions*: Our cases highlight that IgAD can be observed in nephrotic syndrome and nephropathy in children with IgAD appears to be complicated and difficult to treat with corticosteroids alone. Further research is needed to better describe the clinical manifestations and pathological pictures among subjects with IgAD and nephrotic syndrome to understand whether IgAD has a prognostic value in children with nephrotic syndrome and to let clinical physicians define a more personalized and appropriate approach for the management of these patients.

## 1. Background

Immunoglobulin A (IgA) deficiency (IgAD) is defined as a serum IgA level below or equal to 7 mg/dL in subjects older than 4 years and in whom other causes of hypogammaglobulinaemia have been excluded [1]. IgAD is a life-long disorder in most cases, and reports have shown that low IgA levels remain stable in IgAD patients over more than 20 years of observation [2,3]. Although IgAD is the most common form of primary immunodeficiency in Western countries, there is a marked variability in its prevalence in different ethnic groups, suggesting a genetic basis for the disorder [4,5].

IgAD can be acquired as a result of certain medications (e.g., phenytoin, carbamazepine, valproic acid, zonisamide, sulfasalazine, gold, penicillamine, hydroxychloroquine, and nonsteroidal anti-inflammatory drugs) or infections (e.g., Epstein-Barr virus infection, congenital cytomegalovirus infection, congenital toxoplasmosis, congenital rubella, HIV infection) [6]. Moreover, it can be a feature of genetic disorders such as chromosomopathies (e.g., chromosome 18q deletion syndrome, monosomy 22 disease, trisomy 22 or trisomy 8) and monogenic diseases (e.g., ataxia-telangiectasia syndrome, Wiskott–Aldrich syndrome) [6]. IgAD can be sporadic or associated with common variable immunodeficiency (CVID) in approximately 20% of cases [7]. Differences in population prevalence in various ethnic groups, strong familial clustering of both disorders, a predominant inheritance pattern in multiple-case families compatible with autosomal dominant transmission and a high relative risk for siblings suggest the involvement of genetic factors that regulate lymphocyte survival and activation in the pathogenesis of IgAD/CVID [8].

Most affected subjects with IgAD are asymptomatic and are diagnosed during routine tests for other conditions or following screening of a related proband with IgAD/CVID, but some do have problems over time [6,9]. Clinical manifestations can include respiratory and gastrointestinal tract infections, atopy, autoimmune diseases, celiac disease and malignancy. Long-term vigilance is recommended [9]. Up to one-third of symptomatic patients experience recurrent infections, such as viral infections, otitis media and sinopulmonary infections, as well as gastrointestinal infections. In addition to infections, IgAD may also play a role in the development of autoimmune disorders, including lupus-like illnesses, arthritis thyroiditis and type 1 diabetes mellitus; haematologic disorders, including neutropenia and thrombocytopenia; and gastrointestinal illnesses, including Crohn’s disease, ulcerative colitis, and celiac disease [10,11,12]. Patients with IgAD are also at higher risk for gastrointestinal and lymphoid malignancies later in life [1]. There have been several reports on SIgAD complicated by glomerulonephritis in adults, but only very few cases of IgAD with nephropathy have been reported in children. We present two cases of IgAD with relapsing nephrotic syndrome in pediatric age.

## 2. Case Presentation

### Case 1

A 4-year-old boy presented with bilateral periorbital oedema dating back a month and was admitted to our hospital. He had a good general condition and normal pressure values. The results of laboratory tests revealed normal creatinine, hypoprotidaemia (3.8 g/day), hypoalbuminaemia (1.8 g/dL), hypercholesterolaemia (283 mg/dL), hypertriglyceridaemia (242 mg/dL) and nephrotic proteinuria (2.7 g/day < 40 mg/mq/h). Immunological studies showed normal C3 and C4, increased antinuclear antibody titre with mild positivity at IFA Hep-2 (titre of 1:160, speckled pattern), anti-dsDNA antibody negativity, phospholipase A2 receptor (PLA2R) antibodies negativity, IgG 450 mg/dL (less than 2 standard deviations below the normal age-adjusted mean), IgA 3 mg/dL (less than 2 standard deviations below the normal age-adjusted mean) and IgM 94 mg/dL (normal). HBsAg and hepatitis B and C virus serology results were negative, while Epstein-Barr virus, cytomegalovirus and varicella-zoster virus serology results were positive for IgG. Renal ultrasound was normal.

Without performing renal needle biopsy due to ethical issues, a diagnosis of idiopathic nephrotic syndrome associated with IgAD was made, and steroid treatment (prednisone 60 mg/mq/day) was started. Proteinuria became negative after 12 days of treatment; after 4 weeks, the prednisone dose was tapered to 40 mg/mq/day given every other day for 4 weeks, and then this alternate-day dose was slowly tapered over the next 2 months. The subsequent measurement of serum immunoglobulins showed normal IgG and IgM values, but IgA remained very low.

Three months after diagnosis of nephrotic syndrome, during steroid tapering, the child experienced a relapse that was treated with high dose of prednisone (60 mg/mq/day) until remission, then changed to an alternate-day dose for 4 weeks that gradually tapered over three months. During decalage of steroid therapy for the first relapse, the child experienced a second relapse that required a high dose of steroid treatment as before. Subsequently, the child had two additional relapses, becoming frequent and steroid dependent. Therefore, he required rituximab (a chimeric monoclonal anti-CD20 antibody) infusion. Currently, after 2 years his nephrotic syndrome is in remission. His IgA is always very low, with normal values of other immunoglobulins and IgG subclasses.

### Case 2

A 2-year-old boy was admitted to our hospital for bilateral periorbital oedema, scrotal oedema and weight gain. He had a good general condition and normal pressure values. Investigations showed normal creatinine, hypoprotidaemia (3.9 g/day), hypoalbuminemia (2 g/dL), hypercholesterolaemia (417 mg/dL), hypertriglyceridemia (444 mg/dL), nephrotic proteinuria (spot proteinuria/creatininuria 15.5; nephrotic range > 2) and transient microscopic haematuria (5–10 erythrocytes per high-power field). Immunological studies revealed normal C3 and C4, increased antinuclear antibody titre (30.2 U/mL) with mild positivity at IFA Hep-2 (titre of 1:80, speckled pattern), anti-dsDNA antibody negativity, PLA2R antibodies negativity, IgG 42 mg/dL (less than 2 standard deviations below the normal age-adjusted mean), IgA 5 mg/dL (less than 2 standard deviations below the normal age-adjusted mean) and IgM 87 mg/dL (normal). Virus serology was negative, and renal ultrasound was normal.

Without performing renal needle biopsy due to ethical issues, a diagnosis of idiopathic nephrotic syndrome associated with IgAD and partial deficiency of IgG was made, and steroid treatment (prednisone 60 mg/mq/day) was started. The proteinuria decreased, but after 6 weeks of steroid therapy, it did not become negative. Therefore, he was treated with 3 high-pulse doses of methylprednisolone followed by steroid therapy, which achieved remission. As late responder, he started cyclosporin, and then steroid was gradually tapered over one month. While he was on cyclosporin therapy, he relapsed six months later and was treated with steroid therapy, with transitory remission. Therefore, cyclosporin was shifted to tacrolimus after one year. In the next three years, while he was on tacrolimus therapy, he had three relapses that required steroid treatment, and then he was treated with ofatumumab (a fully human monoclonal anti-CD20 antibody) infusion. As he had a relapse nine months later, a second ofatumumab infusion was made.

After 2 years, he is still in remission. During follow-up, the measurement of serum immunoglobulins showed IgAD associated with partial deficiency of IgG and normal values of IgM; coeliac disease antibody tests were negative, but he is HLA-DQ2 positive.

## 3. Discussion

Nephropathy has been previously observed in few children with IgAD (Table 1). Liu et al. described a 6-year-old boy with nephrotic syndrome and a medical history of asthma, chronic otitis media and IgA deficiency (IgA levels less than 3 mg/dL) [13]. A percutaneous renal needle biopsy was performed, and a diagnosis of diffuse and generalized mesangiopathic glomerulonephritis was rendered. Direct IFA showed moderate granular and clumped staining in mesangial areas for IgG, IgM, C3, and Clq. The authors underlined the importance to detect IgAD in patients with renal diseases because they frequently require blood transfusion and may experience anaphylactic reactions. Kawasaki et al. presented a 5-year-old boy with a primary nephrotic syndrome and IgAD (IgA less than 5 mg/dL) [14]. Oral prednisolone (2 mg kg/day) was started, and the proteinuria became negative at after 14 days of treatment. However, the subject experienced a relapse of nephrotic syndrome caused by influenza virus infection after 9 months. Methylprednisolone pulse therapy was then started and resulted in complete remission for 6 months. The patient subsequently experienced one relapse associated with the viral infection. A percutaneous renal needle biopsy was performed at 6 years old. IFA staining showed diffuse granular deposits of IgG, IgG4, and C3 in the glomerular capillary wall. The final pathological diagnosis was diffuse membranous glomerulonephritis stage 1. The authors did not find direct evidence of a pathogenic association between IgAD and membranous glomerulonephritis and supposed that the mechanism of onset of glomerulonephritis in their patient with IgAD could be the association with circulating immune complexes. Ichikawa et al. described a 9-year-old boy with Rasmussen syndrome, IgAD (IgA less than 5 mg/dL) and juvenile alopecia who developed a nephrotic syndrome [15]. The boy died at the age of 14 years, and a kidney specimen indicated progressive membranous nephropathy. The authors considered that the patient suffered from a multimodal autoimmune disorder producing juvenile alopecia, autoimmune encephalitis and a membranous nephropathy, based on the IgAD. In our cases, although renal biopsy was not performed, the diagnosis of idiopathic nephrotic syndrome was clear and made on the basis of clinical and laboratory findings. Both children presented IgAD (the first patient with an IgA level of 3 mg/dL, the second patient with an IgA level of 5 mg/dL) and showed a complicated, relapsing nephrotic syndrome. We do not have an explanation on persistence of partial IgG deficiency in the second patient. Moreover, both children showed the presence of antinuclear antibody titre with mild positivity at IFA Hep-2, but anti-dsDNA antibody were negative. Interestingly, both children needed a personalized therapeutic approach with monoclonal antibodies directed against CD20 in order to obtain a favourable outcome. Due to PLAR2 antibodies negativity, membranous nephropathy can be excluded and positive outcome with anti-CD20 monoclonal antibodies suggest a diagnosis of focal segmental glomerulosclerosis (FSGS). It is difficult to understand whether IgAD is causally linked or of prognostic value in minimal change-FSGS disease. Our speculation is that if IgAD is of prognostic value in these patients, it indicates steroid-dependence or -resistance warranting treatment with anti-CD20 monoclonal antibodies.

## 4. Conclusions

Our cases highlight that IgAD can be observed in nephrotic syndrome and nephropathy in children with IgAD appears to be complicated and difficult to treat with corticosteroids alone. Further research is needed to better describe the clinical manifestations and pathological pictures among subjects with IgAD and nephrotic syndrome (including the meaning of antinuclear antibody positivity) to understand whether IgAD has a prognostic value in children with nephrotic syndrome and to let clinical physicians define a more personalized and appropriate approach for the management of these children.

## Figures and Tables

**Table 1:** Renal pathology in children with IgA deficiency.

Case No.	Age	Sex	IgG	IgM	IgA	Underlying Disease	Kidney Histology	Immunofluorescence	Reference
1	6	M	N	NA	3 mg/dL	Asthma	Diffuse and generalized mesangiopathic glomerulonephritis	IgG, IgM, C3 and C1q	Liu et al., 1995 [13]
2	5	M	N	N	<5 mg/dL	None	Diffuse membranous glomerulonephritis stage I	IgG, IgG4, and C3	Kawasaki et al., 2004 [14]
3	9	M	N	N	<5 mg/dL	Rasmussen syndrome	Stages III-IV of membranous nephropathy	NA	Ichikawa et al., 2009 [15]
4 *	4	M	N	N	3 mg/dL	None	NA	NA	
5 *	2	M	45 mg/dL	N	5 mg/dL	None	NA	NA	

M: male; NA: not available; N: normal value; *: case described in the present study.
